# Small cell neuroendocrine carcinoma with adenocarcinoma and high grade squamous intraepithelial neoplasia of the cervix

**DOI:** 10.4322/acr.2023.452

**Published:** 2023-11-13

**Authors:** Guralarasan Gurubalan, Amber Parwaiz, Surabhi Ajit, Tarun Kumar, Madhu Kumari, Punam Bhadani

**Affiliations:** 1 All India Institute of Medical Sciences, Patna, Bihar, India

**Keywords:** Adenocarcinoma, Human Papillomavirus Viruses, Neuroendocrine Tumors, Squamous Intraepithelial Lesions

## Abstract

Neuroendocrine neoplasm (NEN) of the cervix is a malignant tumor and is classified into low and intermediate-grade neuroendocrine tumor (NET), and high-grade small cell neuroendocrine carcinoma (SCNEC), and large cells neuroendocrine carcinoma (LCNEC). SCNEC of the cervix is an Infrequent tumor with an incidence of less than 1% of all gynecological malignancies. It is characterized by small to medium-sized tumor cells with scant cytoplasm and neuroendocrine differentiation. Most cases of SCNEC of the cervix manifest in pure forms, and only cases show coexisting, non-neuroendocrine component of HPV-associated adenocarcinoma or squamous cell carcinoma. In this report, reviewing the literature, we present one such unique case of SCNEC of the cervix with adenocarcinoma and high-grade squamous intraepithelial neoplasia.

## INTRODUCTION

Neuroendocrine neoplasms (NEN) are malignant tumors arising from neuroendocrine cells commonly found in the gastrointestinal tract, pancreas, and lungs.^1^ These cells are of fetal neuroectodermal origin and have an immunohistochemical profile similar to endocrine cells.^2^ Due to their endocrine nature, NENs may be able to secrete hormones and are therefore called functional, whereas NENs that do not secrete are considered non-functional.^2^ According to WHO 2020 genital tract, neuroendocrine neoplasms involving the female genital tract except the ovary are categorized into Neuroendocrine tumors (NET), small cell neuroendocrine carcinomas (SCNEC), large cell neuroendocrine carcinomas (LCNEC) and non-neuroendocrine carcinoma admixed with neuroendocrine carcinoma.^3^ Approximately 1% of all cervical cancers are attributed to small-cell neuroendocrine carcinoma.^3^ Most of these cases manifest as pure forms, while only 4% of tumors are linked to coexisting adenocarcinoma.^3^ Among cervical malignancies, adenocarcinoma comprises 5% of cases, while neuroendocrine neoplasms (NEN) are infrequent, making up less than 1% of all gynecological malignant tumors. Carcinoma, mixed with neuroendocrine carcinoma, is even more uncommon.^3^ Nearly all cases of cervical cancer are linked to the presence of the human papillomavirus (HPV), which is detected in an overwhelming 99.7 percent of cervical cancer cases.^4^ Squamous cell carcinoma and adenocarcinoma are the predominant histologic subtypes of cervical cancer, accounting for approximately 70 percent and 25 percent of cases, respectively.^5^ Cervical neuroendocrine carcinomas are frequently linked to the Human Papilloma Virus, which is also believed to play a role in the progression of the tumor.^6^ The average recurrence-free and overall survival has been calculated to be approximately 16 months and 48 months, respectively.^7^ On extensive literature search on PubMed, Medline, and Scopus, we found a total of 20 cases of Neuroendocrine neoplasm with Non-neuroendocrine carcinoma reported to date and only one case of this unique combination of Neuroendocrine neoplasm with adenocarcinoma and high-grade squamous intraepithelial lesion (HSIL). This article presents a highly uncommon and distinctive case involving a mixed malignant tumor discovered in the cervix. The tumor comprises three distinct components: neuroendocrine carcinoma, adenocarcinoma, and HSIL. This extraordinary combination distinguishes this case from others, making it a noteworthy case for additional investigation.

## CASE REPORT

**Figure 1:**
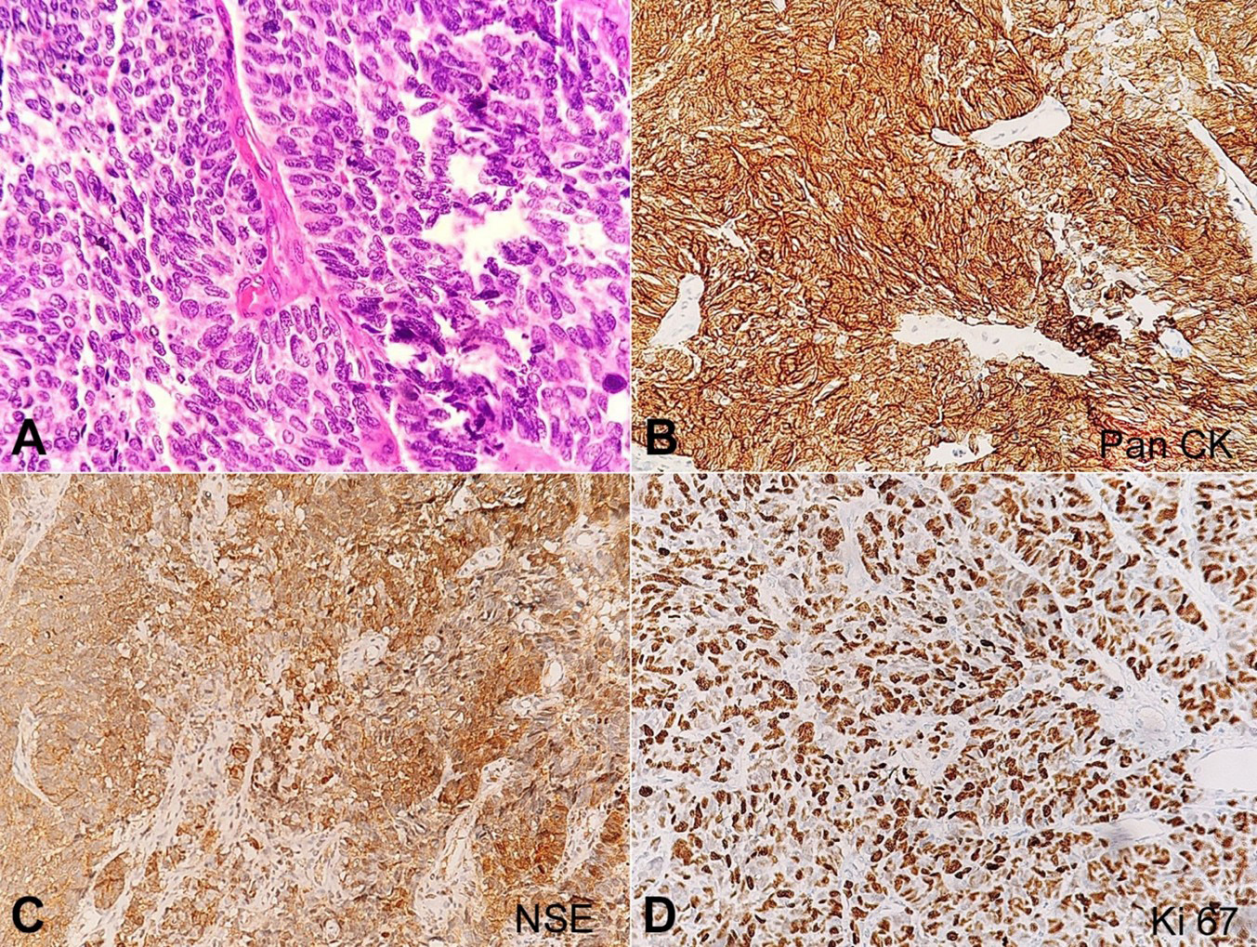
Photomicrographs of the cervical biopsy. **A –** Tumor arranged in sheets and nest separated by thin fibrovascular septa. Tumor cells show moderate pleomorphism, hyperchromatic nuclei, round to ovoid nuclei, inconspicuous nuclei and scant to moderate cytoplasm (H&E 400x); **B –** On IHC, tumor cells are positive for PanCK (100x); **C –** NSE (100x); **D –** High Ki-67 proliferation index (100x).

A 75-year-old female patient presented with complaints of vaginal bleeding persisting for one month, accompanied by lower abdominal pain. Physical examination revealed a cervical growth measuring approximately 2.2x2 cm. A cervical biopsy showed a tumo arranged in sheets and nests, separated by fibrovascular septa. The tumor cells exhibited moderate nuclear pleomorphism, hyperchromatic round to ovoid nuclei, inconspicuous nucleoli, and scant cytoplasm (Figure 1A). Mitotic activity was 15 to 20 mitotic figures per 10 high-power fields. Immunohistochemistry (IHC) yielded positive results for pancytokeratin (PanCK) (Figure 1B), Non-Specific Enolase (NSE) (Figure 1C), Synaptophysin, and high ki-67 proliferation index (Figure 1D) while negative for Chromogranin, p40, p63, and vimentin. Based on the histopathological and IHC findings, a diagnosis of small-cell neuroendocrine carcinoma was rendered.

**Figure 2:**
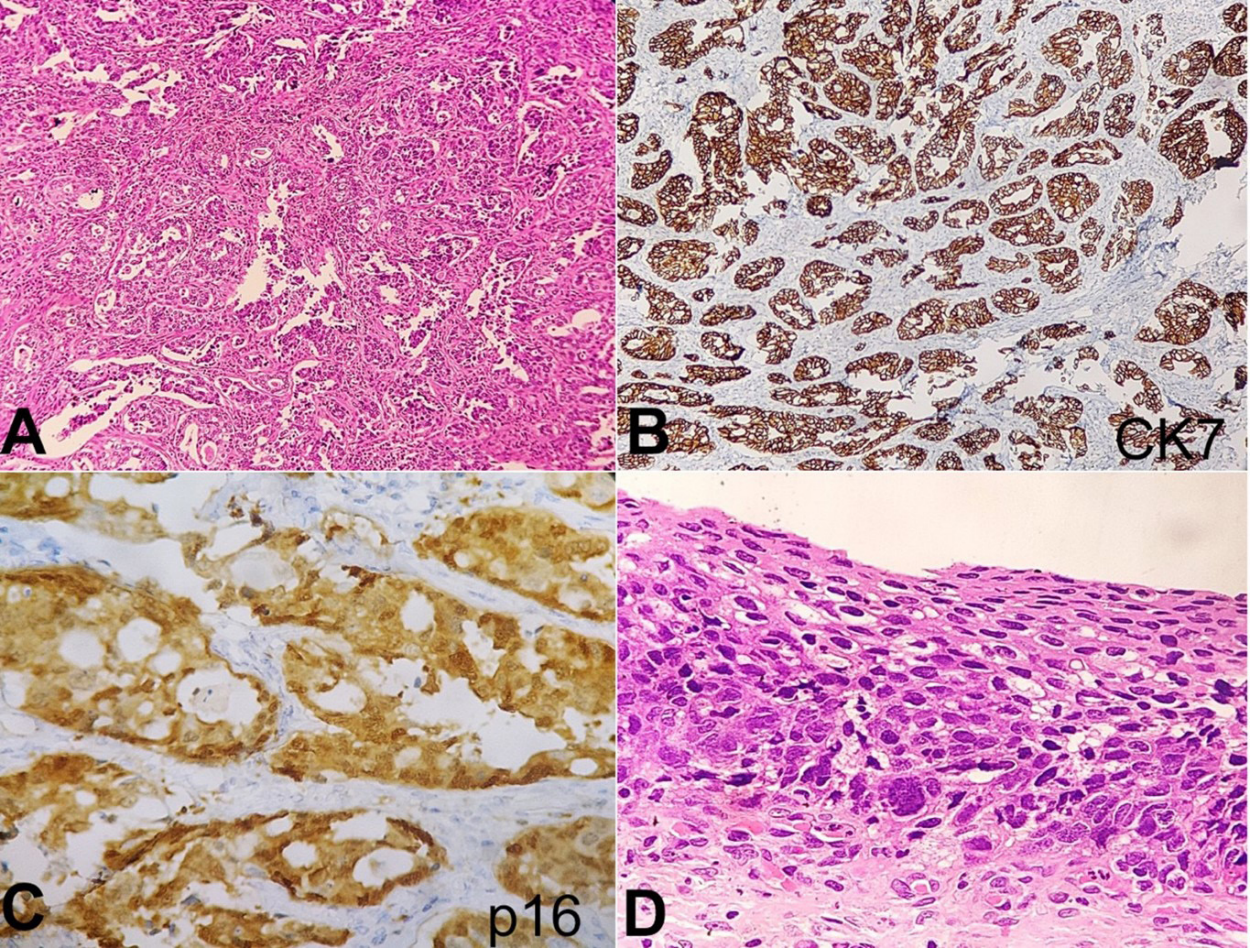
Photomicrograph of the Cervix. **A –** Tumor cells are arranged in infiltrative glands having vesicular chromatin and a moderate amount of cytoplasm (H&E 100x); **B –** CK7 staining in adenocarcinoma component (100x); **C –** Diffuse p16 staining (400x); **D –** Overlying squamous lining shows HSIL (H&E 400x).

The patient underwent a radical hysterectomy. Gross examination revealed a circumferential grey-white growth, measuring 2.2x2x1.5cm, involving the cervix and obliterating the endocervical canal. The microscopy revealed a collision tumor. One tumor component was arranged in sheets and nests, consisting of round to ovoid cells with high nuclear-to-cytoplasmic ratios and a scant amount of cytoplasm, indicative of neuroendocrine differentiation. Other components consist of an infiltrating tumor arranged in a glandular pattern exhibiting moderate pleomorphism, vesicular chromatin, and a moderate amount of cytoplasm consistent with adenocarcinoma morphology. IHC showed positive staining for CK7 in the adenocarcinoma component (Figure 2A-B). Furthermore, the overlying squamous lining showed high-grade squamous intraepithelial lesion (HSIL) (Figure 2D). Diffuse p16 staining (Figure 2C) was observed in both the adenocarcinoma component and overlying dysplastic squamous epithelium.

Following a radical hysterectomy, the patient's treatment plan was initiated with a course of chemotherapy comprising etoposide and cisplatin. Notably, the patient has exhibited satisfactory progress and a favorable medical status throughout the subsequent four-month period.

## DISCUSSION

In 1972, the initial documentation of cervical neuroendocrine neoplasms (NENs) was provided by Albores-Saavedra et al.^8^ The specific origin of cervical neuroendocrine neoplasms (NENs) needs to be fully understood.^9^ Nonetheless, it is believed that argyrophilic cells present in the epithelium of both the ectocervix and endocervix may serve as a potential precursor for developing NENs.^9^ SCNEC is the predominant subtype among cervical neuroendocrine neoplasms (NENs), accounting for approximately 80% of the cases.^10,11^ It represents less than 1% of all female reproductive system malignancies .^10^ Only a limited number of cases involving the cervix’s neuroendocrine neoplasms (NEN) have been documented to exhibit coexisting adenocarcinoma. This case, characterized by the coexistence of adenocarcinoma and neuroendocrine carcinoma, was called mixed adeno-neuroendocrine carcinoma.^12^ Each component, adenocarcinoma and neuroendocrine carcinoma, constitutes more than 30% of the overall tumor.^12^ The primary type of tumor observed is a small cell neuroendocrine tumor (NET), predominantly found in its pure form. However, approximately 4% of cases are linked to adenocarcinoma.^13^ Clinically, the cervix’s neuroendocrine neoplasms (NEN) manifest as symptoms such as vaginal bleeding after menopause, abnormal vaginal discharge, spotting after sexual intercourse, and the presence of a cervical mass (Table 1).^13^

**Table1:** Reported cases of neuroendocrine neoplasm with non-neuroendocrine carcinoma

Ref	# of Cases	Age (Y)	Neuroendocrine tumor	Non-neuroendocrine carcinoma	HPV status
^17^	1	57	LCNEC	Adenocarcinoma	p16 positive
^18^	1	42	SCNEC	Adenocarcinoma and HSIL	p16 positive
^19^	1	56	NEC	Adenocarcinoma	p16 positive
^20^	1	36	SCNEC	Squamous cell carcinoma	p16 positive
^21^	1	65	SCNEC	Adenocarcinoma in situ	p16 positive
^22^	1	59	NEC	Adenocarcinoma	p16 positive
^23^	2	35, 38	NEC	Adenocarcinoma	p16 positive
^24^	1	34	LCNEC	Signet ring cell carcinoma	HPV16/18 by ISH
^25^	1	31	LCNEC	Mucinous adenocarcinoma	p16 positive
^26^	10	25-48	NEC	Adenocarcinoma (7 cases)	p16 positive
				Squamous cell carcinoma (2 cases)	
				Adenosquamous cell carcinoma (1 case)	

Y= years

In our case, the patient presented with vaginal bleeding. The age of presentation in our patient, who is 75 years old, stands out as unusual compared to the typical age range of 25 to 65 years observed in most reported cases (Table 1) involving mixed neuroendocrine carcinoma (NEC) with other carcinoma. The fact that the patient's age at presentation is atypical adds uniqueness to our findings. Diagnosing cases that involve both neuroendocrine and non-neuroendocrine tumors simultaneously can pose a challenge. Neuroendocrine tumors (NETs) display positive staining for specific markers, including synaptophysin (SNP), chromogranin (CG), CD56 (N-CAM), and neuron-specific enolase (NSE).^8^ At least two markers are required to confirm a NET diagnosis, with SNP and CD56 being the most sensitive.^8^ Research studies have indicated that approximately 60% of cases show negative staining for synaptophysin and chromogranin, while about 33% display negative staining for neuron-specific enolase.^8^ Our case exhibited positive staining for both synaptophysin and NSE, confirming neuroendocrine differentiation. Conversely, adenocarcinoma is an invasive epithelial tumor characterized by atypical cells exhibiting glandular differentiation and distinct features such as hyperchromatic nucleus and pleomorphism. Additionally, the identification of mitotic figures and infiltration into the cervical stroma further supports the diagnosis of adenocarcinoma.^14^ The most frequently observed non-neuroendocrine component is adenocarcinoma of the typical HPV-associated type. On the other hand, the neuroendocrine component is typically represented by neuroendocrine carcinomas.^3^ Similar to cervical squamous cell carcinomas and adenocarcinomas, most high-grade neuroendocrine neoplasms of the cervix have been found to contain high-risk human papillomavirus (HPV) DNA^15^ Specifically, the HPV-16 and HPV-18 subtypes are commonly detected in these cases.^15^ In our case, the neoplastic component of adenocarcinoma and high-grade squamous intraepithelial lesion (HSIL) showed immunohistochemical expression of p16, suggesting the role of HPV in pathogenesis. HSIL is characterized by abnormal squamous cells and is closely associated with human papillomavirus (HPV) infection. It's important to note that although not all HSIL cases will develop into cancer, HSIL itself is recognized as a precancerous lesion. As a result, aggressive treatment approaches are typically employed to manage HSIL.^16^ Among the different histological variants, NEC is the most aggressive type, with 5-year survival rates as low as 35%, highlighting its more unfavorable prognosis than other types. Recurrences are predominantly observed within the initial 2-year period following the diagnosis.^7^ Given the uncommon nature of this form of cervical malignancy, there is currently no established personalized treatment regimen specifically tailored for pure neuroendocrine carcinomas or mixed neuroendocrine non-neuroendocrine neoplasms. Clinicians typically adopt multimodal strategies that draw upon treatment protocols utilized for neuroendocrine carcinomas of the lung.^13^ The Society of Gynecologic Oncology (SGO) has recently formulated guidelines for women with cervix neuroendocrine carcinomas, suggesting a multimodal approach involving neoadjuvant chemotherapy based on etoposide and platinum agents.^13^

## CONCLUSION

Given a common causal relationship between HPV infection and cervical tumors, coexisting lesions of cervical and glandular lesions (in situ and invasive) are not so uncommon. However, this case represents a unique cervical tumor encompassing small-cell neuroendocrine carcinoma, adenocarcinoma, and HSIL. Combining these components adds to the case's complexity and highlights the importance of thorough sampling and pathological examination to demonstrate each component in a resected specimen. Further research and studies are warranted to understand better the pathogenesis, prognosis, and optimal treatment approaches for this uncommon condition.
